# Decoding the Modulation of Temperature During Withering on the Flavor Quality and Appearance of Taiping Kuihong Black Tea

**DOI:** 10.3390/foods15112027

**Published:** 2026-06-05

**Authors:** Xuefei Peng, Xin Huang, Jihan Zhang, Ruoyu Chen, Wuji Yu, Yuxuan Zhang, Tiehan Li, Jingming Ning, Shaode Hu

**Affiliations:** 1 School of Tea Science, Anhui Agricultural University, Hefei 230036, China; 13965521468@163.com (X.P.); 17755611105@163.com (X.H.); 15966386951@163.com (J.Z.); 15105518742@163.com (R.C.); 18955675981@163.com (W.Y.); 18740704892@163.com (Y.Z.);; 2 State Key Laboratory of Tea Plant Germplasm Innovation and Resource Utilization, Hefei 230036, China

**Keywords:** Taiping Kuihong, freeze-withering, theaflavins, sensory quality, volatile substances

## Abstract

This study introduced freeze-withering to resolve the conflict in traditional Taiping Kuihong black tea processing, where rolling enhances flavor but damages the integrity of the leaf shape. Using sensory evaluation, high-performance liquid chromatography, and gas chromatography–mass spectrometry, the effects of three freezing treatments (−20 °C, −80 °C, and liquid nitrogen at −196 °C) were analyzed and compared with traditional withering (CK). The results demonstrated that low-temperature treatments better preserved the morphological integrity of ‘two leaves and one bud.’ Compared to the CK group, the −20 °C low-temperature treatment group had a significantly higher proportion of esters among the volatile compounds in Taiping Kuihong black tea. Key floral fragrance components were abundant, while grassy undertones were greatly decreased. Concurrently, this group had the highest levels of theaflavins and TF/TR ratios. The −80 °C treatment was highly effective in preserving thearubigins; however, it resulted in a less complex fragrance profile with a reduced theaflavin level. Liquid nitrogen treatment caused aberrant accumulation of theabrownins, poor aroma harmony, and the lowest theaflavin level. This study indicates that −20 °C freezing withering is the best method for harmonizing the appearance and internal quality of Taiping Kuihong, giving critical evidence for targeted processing of unique black teas.

## 1. Introduction

Black tea is one of the most consumed types of tea globally, and its quality is determined by factors such as appearance, aroma, and taste [1,2,3]. While the perfume is produced by a complex mixture of volatile substances, the look is shaped by the physical structure of the leaves during the rolling process. Alcohols and terpenes like linalool, geraniol, and β-ionone are frequently linked to pleasant aromas like floral, fruity, and sweet notes, while C6 aldehydes and alcohols like linalool and n-hexanal are mainly responsible for grassy notes [4,5]. The composition and proportions of non-volatile pigments like flavones and arubigins are closely linked to the color and flavor of liquor. A higher theaflavin content and TF/TR ratio result in a brighter, golden liquor color and a fresher, more invigorating flavor [6]. Conversely, excessive accumulation of theabrownins leads to a darkened liquor color and a weak, insipid flavor [7,8]. Taiping Kui black tea is a variety of black tea produced in Huangshan City, Anhui Province, China, made from the fresh leaves of the Shida tea plant [9,10]. Withering, as the first and crucial stage in the processing of black tea, has a profound impact on the subsequent rolling and fermentation stages, as well as on the final quality of the tea [11]. Traditional withering methods essentially encourage physicochemical changes in fresh leaves by controlling temperature, humidity, and airflow velocity [12,13]. This must be followed by extensive mechanical rolling to break cells and activate enzymatic oxidation processes. However, the severe rolling used in traditional procedures to accomplish sufficient cell rupture frequently leads in tightly wrapped strands or even the separation of buds and leaves, which reduces morphological distinctiveness and undermines value foundations. To address this constraint, this study investigated using physical pretreatment technologies to replace or minimize mechanical rolling intensity. Research into the application of freezing technology in black tea processing has been ongoing for many years. Early studies revealed that low-temperature treatment can affect the activity of key enzymes such as polyphenol oxidase [14], and has a significant impact on the quality of Gongfu black tea [15,16]. However, current research on freeze-withering has largely focused on the independent analysis of intrinsic indicators, with a lack of an integrated perspective that combines sensory evaluation and metabolomics; as a result, the overall mechanisms by which freeze-withering shapes the flavor profile of black tea have yet to be systematically understood. More importantly, the existing studies’ rolling methodologies continue to rely on standard parameters, with no adjustments adapted to the properties of leaves after freeze-pretreatment. Furthermore, no studies have been conducted to actively mold specific visual styles (such as sharp points) using a mix of gentle rolling and pinching approaches. A systematic strategy using freeze-withering to optimize both the visual appearance and overall flavor profile of Taiping Kuihong black tea has yet to be studied [15,16,17,18,19].

Aroma is a key component of black tea’s flavor profile, defining its sensory quality and market competitiveness [20,21,22], and its production and evolution are especially sensitive to external stresses. According to research, adequate freeze-withering can improve the freshness and sweet aroma of brewed liquor [17,23,24]. However, other investigations have found that badly regulated freezing methods might result in the loss of particular unique scent components or modifications in the overall aroma profile [18,19]. It is particularly worth noting that, whilst this study incorporates gentle kneading to complement cryomoulding, the reduced intensity of the kneading process implies a change in the extent of cell damage. This will directly affect the release flux and conversion rate of aroma precursors during enzymatic oxidation, potentially influencing the final aroma composition and profile in ways that differ from those of traditional processes. The formation and accumulation of aromatic compounds are more susceptible to interference from temperature, the extent of cell rupture and subsequent enzymatic reactions, and the mechanisms underlying these changes are also relatively complex [14,16]. Consequently, whilst exploring the synergistic shaping effects of freeze-withering and gentle rolling, systematically elucidating the interrelationships between ‘freeze-withering—gentle rolling—aroma composition’ is of crucial importance in ensuring that innovations in appearance do not come at the expense of intrinsic quality, and in achieving a genuine synergistic optimization of both appearance and flavor.

Accordingly, this study used traditional processing methods as a control and established three freeze-wilting treatment protocols at −20 °C, −80 °C and with liquid nitrogen. Subsequent processing steps were standardized across all the treatments. By combining sensory evaluation with metabolomic analysis, the study systematically examined the synergistic changes in the appearance, taste and aroma of Taiping Kuihong black tea resulting from the combined effects of different freezing intensities and light rolling. This study represents a preliminary exploration of methods for the co-design of freeze-wilting and light rolling processes, with the aim of actively shaping the ‘pointed’ appearance of Taiping Kuihong black tea. The findings will not only provide a processing protocol for Taiping Kuihong that balances appearance and flavor quality, but they will also reveal the differential effects of varying freeze-withering temperatures under light rolling conditions on the intrinsic properties of black tea, providing new data and insights to refine the theoretical framework for freeze-wilting and rolling optimization.

## 2. Materials and Methods

### 2.1. Materials and Reagents

The experimental materials were fresh leaves of the Persimmon Big Leaf tea tree variety, harvested in April 2025 from the Wan Nan Comprehensive Experimental Station (30.325° N, 118.005° E) of Anhui Agricultural University in Huangshan District, Huangshan City, Anhui Province, China, according to the one bud, two or three leaves standard.

Methanol, acetic acid, and acetonitrile (all HPLC-grade) were obtained from Shanghai Aladdin Bio-chemical Technology Co., Ltd., Shanghai, China; sodium carbonate, Folin–Ciocalteu reagent, gallic acid, dipotassium hydrogen phosphate, disodium hydrogen phosphate, sodium dihydrogen phosphate dihydrate, ninhydrin, hydrochloric acid, decyl acetate, TF standard, and n-alkane mixed standard (C8–C40) were obtained from Sigma-Aldrich, St. Louis, MO, USA.

### 2.2. Experimental Treatment

#### 2.2.1. Tea Sample Preparation

Fresh leaves consisting of one bud and two leaves of the ‘Shidacha’ variety were used as raw material. For each treatment, 150 g of fresh leaves were taken and replicated three times independently.

Freezing treatment group: The fresh leaves were first withered in a withering ma-chine (air temperature 35 °C) until the moisture content reached approximately 65%, then divided into three equal portions and subjected to the following freezing treatments: (1) −20 °C group: frozen for 4 h in a −20 °C freezer; (2) −80 °C group: frozen for 4 h in a −80 °C freezer; (3) liquid nitrogen group: the withered leaves were placed in a stainless steel container, and liquid nitrogen (−196 °C) was added to ensure thorough contact, followed by freezing for 10 min. After thawing, surface moisture was gently blotted from the tea samples using absorbent paper, followed immediately by light rolling for 5–10 min. The rolling machine used was the YX701 tea withering machine, manufactured by Xiaodong Yaxing Instrument Co., Ltd., Yuyao City, Zhejiang Province, China. Following rolling, the samples were fermented for 4 h at 28 °C and 90% relative humidity. Upon completion of fermentation, the tea leaves were straightened by hand, and a tip-pinching machine was used to wrap the two leaves around the tender bud, forming the characteristic ‘two leaves embracing one bud’ shape. The shaped tea leaves were neatly spread out on a wire mesh tray lined with cotton cloth, placed on a specially designed shaping machine, covered with cotton cloth, and lightly pressed into shape using a pneumatic roller. Finally, the tea was subjected to initial drying (100 °C) until 90% dry, then cooled and subjected to final drying (60 °C, 30–60 min) until fully dry.

**Figure 1:**
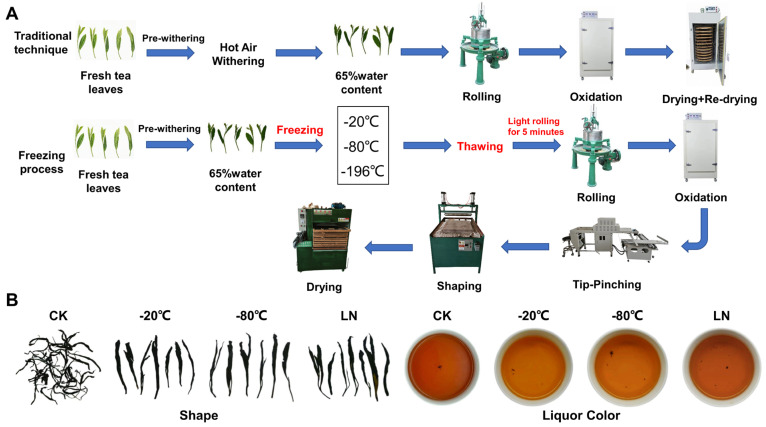
Appearance and liquor color of Taiping Kuihong tea (**A**); Flow chart of the traditional processing and freezing withering processes (**B**). Note: The abbreviations shown in the figure refer to the −20 °C, −80 °C, and liquid nitrogen (−196 °C, LN) treatments, and traditional withering (CK).

Control group: Fresh leaves were withered in a withering machine for 7 h (air temperature 35 °C) until the moisture content reached approximately 65%; without undergoing freezing treatment, they were directly subjected to traditional heavy rolling for 90 min. The subsequent fermentation (28 °C, 90% RH, 4 h) and drying (initial drying at 100 °C until 90% dry, followed by final drying at 60 °C for 30–60 min until fully dry) processes were identical to those of the frozen treatment group, but the shaping steps of rolling into strips and pinching the tips were omitted. See the flowchart in (Figure 1A).

#### 2.2.2. Sample Collection

All the finished tea samples were sealed, protected from light and stored at 4 °C pending analysis. Each treatment was replicated three times independently, with 100 g of fresh leaves used for each replicate. Subsequent sensory evaluation and physicochemical analysis were conducted using the three biological replicates.

### 2.3. Determination Method

#### 2.3.1. Sensory Evaluation

The study was carried out in a standardized sensory evaluation room by three evaluation experts (one woman and two men) and 12 participants who had received three months of systematic aroma assessment training in accordance with the national standard GB/T 18797-2012 [25]. The three evaluation experts had over ten years’ experience in assessing and scoring tea quality. As the core of the evaluation panel, they were responsible for leading the evaluation process, calibrating the definitions of sensory attributes and verifying the final scores. Twelve postgraduate students participated in the evaluation and scoring of this experiment alongside the experts to ensure the statistical significance and reliability of the results. In line with ISO 8586:2023 [26], all 15 members of the evaluation panel were screened and trained on taste stimulus identification and intensity scoring exercises to ensure a consistent understanding of attribute definitions and the intensity scale. Before testing, each assessor gave written informed consent. According to national policy, sensory analysis does not require the approval of a human ethics committee. These fifteen professionals evaluated the samples’ appearance, liquor color, aroma, flavor and leaf base. The samples of Taiping Kuihong black tea, processed in different ways, were carefully weighed (3 g) and placed in standard tasting cups, to which boiling water (100 °C, 150 mL) was added. After steeping for 4 min, the tea liquor was filtered into tasting bowls for evaluation. The assessors provide comments and scores for each sample; for the dry tea, they evaluated its shape, tenderness, color, uniformity, and purity; the tea liquor was assessed for its color type, hue, brightness, and clarity; the aroma was assessed for its type, intensity, purity, and persistence; the flavor was assessed for its strength, body, smoothness versus astringency, purity versus foreign notes, and freshness versus dullness; and the tea leaves after brewing were evaluated for the scoring system is as follows: appearance 25%, scent 25%, liquor color 10%, flavor 30%, and leaf base 10%. The grading criteria and weighting mechanism were compliant with Chinese National Standards GB/T 23776-2018 and GB/T 14487 [27,28].

Note: For detailed methodological procedures, please refer to the relevant standards [25,26,27,28].

#### 2.3.2. Determination of Physicochemical Composition

Ground tea samples were prepared and their moisture content determined in accordance with GB/T 8303-2013 [29]; the dry matter content was calculated from the difference in mass before and after drying. The free amino acid content was determined in accordance with GB/T 8314-2013 [30] by measuring the absorbance at a wavelength of 570 nm and quantifying it against a standard curve; the tea polyphenol content was determined in accordance with GB/T 8313-2018 [31], using the Folin–Ciocalteu method with gallic acid as the standard to establish a quantitative curve, referencing the methodology of previous research [32]; caffeine, gallic acid, epigallocatechin, epigallocatechin gallate, epicatechin and gallocatechin gallate were determined using high-performance liquid chromatography (HPLC) in accordance with GB/T 30483-2013 [33]; the content of theaflavins, thearubigins and theabrownins was determined using NY/T 3675-2020 [33,34] ‘Determination of Thearubigins and Theabrownins in Black Tea’ (spectrophotometric method) and high-performance liquid chromatography (HPLC) GB/T 30483-2013; and the content of total soluble sugars was determined using the anthrone colorimetric method [35]. The sample extract reacted with anthrone reagent under heated conditions to form a blue-green complex; the absorbance was measured at 620 nm using a spectrophotometer, and quantification was performed using a glucose standard curve.

Note: For detailed methodological procedures, please refer to the relevant standards [29,30,31,33,34,35].

#### 2.3.3. HPLC Conditions

Determination was carried out using ultra-high-performance liquid chromatography (HPLC), specifically an Ultimate 3000 HPLC system (Dionex, Sunnyvale, CA, USA) equipped with an ACQUITY UPLC^®^ BEH Shield RP18 column (2.1 mm × 50 mm, 1.7 μm) from Waters Corporation, Taunton, MS, USA; the column temperature was set at 40 °C and the injection volume at 1 μL. Under these conditions, four theaflavin compounds (TF, TF-3-G, TF-3′-G, TFDG), caffeine and six catechin monomers were separated and quantified simultaneously.

#### 2.3.4. Headspace Solid-Phase Microextraction–Gas Chromatography–Mass Spectrometry (HS-SPME-GC-MS) Analysis of Aromatic Compounds

Sample preparation and extraction: A total of 3 g of tea sample was weighed into a 250 mL conical flask and infused for 5 min using a tea-to-water ratio of 1:50 (g/mL) in accordance with national standards. After the tea infusion was filtered, it was cooled rapidly in an ice-water bath, then 10 mL of the mixed tea infusion was pipetted into a headspace vial. A total of 5 μL of ethyl decanoate internal standard and 3 g of sodium chloride were added and a PDMS magnetic stirrer rotor was used to vortex in order to mix thoroughly. The headspace vial was stabilized in a 40 °C water bath for 15 min. After aging a 50/30 μm DVB/CAR/PDMS extraction fiber for 5 min at 250 °C, it was inserted into the headspace vial and allowed to adsorb for 40 min in a 40 °C water bath.

GC-MS analysis: Upon completion of extraction, the extraction head was immediately inserted into the injection port of a 7890B gas chromatograph–5977B mass spectrometer (Agilent, Santa Clara, CA, USA) and desorbed at 250 °C for 5 min. Chromatographic separation was performed using an HP-5MS capillary column (30 m × 0.25 mm, 0.25 µm), with helium as the carrier gas, a constant flow rate of 1.0 mL/min, and a split ratio of 3:1. Temperature program: hold at 40 °C for 5 min, ramp up to 180 °C at 4 °C/min, then ramp up to 280 °C at 15 °C/min and hold for 5 min. Mass spectrometry conditions: Electron impact (EI) ion source, electron energy 70 eV; ion source temperature 230 °C, quadrupole temperature 150 °C; transfer line temperature 300 °C; mass scan range *m*/*z* 35–500; solvent delay 0 min.

#### 2.3.5. Qualitative and Quantitative Analysis of Aroma

Qualitative analysis: The mass spectra corresponding to each chromatographic peak were searched against the NIST (National Institute of Standards and Technology) standard spectral library, with a match threshold of ≥80% used as the basis for preliminary identification [35]. On this basis, the retention indices (*RI*) of each analyte were calculated using the retention times of a mixed standard of n-alkanes (C8–C40) according to the formula, and the calculated values were compared and verified against the *RI* values reported in the literature for those compounds. Finally, the structures of the compounds represented by each chromatographic peak were confirmed by referring to the published literature on tea aromas. The formula for calculating the *RI* is as follows:

$$RI=100n+\frac{100(t_{x}-t_{n})}{t_{n+1}-t_{n}}$$

where $t_{x}$ is the retention time (min) of the analyte *x*; $t_{n}$ is the retention time (min) of an n-alkane containing *n* carbon atoms; $t_{n+1}$ is the retention time (min) of an *n* + 1-alkane containing *n* + 1 carbon atoms [36].

Quantitative analysis: Volatile components were quantified using the internal standard method. The concentration of each compound of interest was calculated using the following equation, based on the ratio of the peak areas of the compound of interest to the internal standard and the known concentration of the internal standard:

$$C_{i}=\frac{A_{i}\times C_{IS}}{A_{IS}}$$

where $C_{i}$ is the concentration of compound *i* (μg/mL); $A_{i}$ is the peak area of compound *i*; $C_{IS}$ is the concentration of the internal standard (μg/mL); $A_{IS}$ is the peak area of the internal standard.

### 2.4. Data Processing

Orthogonal partial least squares discriminant analysis (OPLS-DA) was performed using SIMCA 14.1 software to calculate variable importance in projection (VIP), whilst simultaneously extracting and visualizing the results of principal component analysis (PCA). In conjunction with SPSS 24.0 software, one-way analysis of variance (ANOVA) was performed to screen for significant aroma components using the criteria *p* < 0.05 and VIP ≥ 1. The calculation of Odor Activity Values (OAV) followed the method described by Xu Mengting et al. [37,38].

The experiment was repeated three times, with results expressed as $\bar{x}$ ± s. One-way analysis of variance (ANOVA) and Duncan’s multiple range test were performed using Excel Office 2020 and SPSS Statistics 24. Statistical analysis and graphing were carried out using SIMCA 14.1, OriginPro 2021 and TBtools v1.113. For the quantitative analysis of volatile compounds, the method described by Shao Shuxian et al. was followed [39,40].

## 3. Results

### 3.1. Effects of Different Freeze-Withering Temperature Treatments on the Sensory Quality of Taiping Kuihong Black Tea

The results of the sensory evaluation indicate that for Taiping Kuihong red tea of the same raw material grade, certain sensory qualities (appearance, liquor color, aroma and flavor, *p* < 0.05) showed significant differences following different withering processes (Table 1). CK stood out in terms of aroma and soup color, with a pure sweet aroma and an orange-red soup color; on the other hand, the freeze-withering method performed better in terms of appearance, with intact bud and leaf shapes. Further comparison revealed that the −20 °C treatment group performed best in terms of flavor harmony, showing little difference compared to CK. Although LN had an orange-yellow broth and a rich taste, it had a noticeable off-flavor, affecting the overall quality (Figure 1).

### 3.2. Effects of Different Freeze-Withering Temperature Treatments on the Content of Main Theaflavins and Other Key Quality Components in Taiping Kuihong Black Tea

#### 3.2.1. The Impact on Theaflavin Content

The content and ratio of theaflavins (TF), thearubigins (TR), and theabrownins (TB) are core chemical indicators that determine the color, taste, and quality grade of black tea [41]. This study analyzed the effects of three freezing treatments (−20 °C, and −80 °C, liquid nitrogen) and CK on these components, with the results shown in Table 2.

As shown in Table 2, different freezing treatments significantly affected the content of the three tea pigments (*p* < 0.05). Theaflavins (TF) are the key component in forming the ‘golden ring’ and the fresh taste of black tea. The results indicate that low-temperature treatment significantly altered the pigment composition characteristics of Taiping Kuihong tea, and there were significant differences in the regulatory directions of different treatments. Among them, the −20 °C treatment significantly increased the TF content and the TF/TR ratio, and this ratio, as a core indicator for measuring the freshness and astringency of black tea, directly enhances the freshness and brightness of the tea liquor. In contrast, while the −80 °C treatment can significantly promote the formation of TR, excessive TR easily led to a bitter taste in the tea soup, which may negatively affect its sensory quality. The LN treatment, on the other hand, significantly increases the TB content, and the accumulation of TB is usually associated with a dull color and bland taste of the tea soup. This treatment may accelerate the over-oxidation of tea polyphenols, which is not conducive to maintaining quality. Compared to CK, the −20 °C low-temperature treatment demonstrated superior quality control effects, while CK still has significant potential for improving freshness.

#### 3.2.2. The Impact on Non-Volatile Components

From Table 3 and Table 4 it can be seen that the content of various non-volatile substances in the processed tea after different freezing methods changed to varying degrees. In terms of tea polyphenol content, the order was ‘−20 °C > −80 °C > liquid nitrogen,’ with the LN tea sample having a tea polyphenol content of 9.99 mg/g, which is close to the CK treatment (9.94 mg/g). The tea polyphenol content of the −20 °C treatment tea sample (11.10 mg/g) and the −80 °C treatment tea sample (10.75 mg/g) is higher than that of the CK level, confirming that freezing treatment had a differential impact on the retention or conversion of tea polyphenols. The soluble sugar content shows a similar trend, with the soluble sugar content in the tea samples treated with liquid nitrogen, −20 °C, and −80 °C being 1.85 mg/g, 1.89 mg/g, and 1.98 mg/g, respectively, all lower than the CK group at 2.94 mg/g. This indicates that the freezing process may have inhibited the accumulation of soluble sugars or promoted their conversion, but the overall difference is smaller than that of tea polyphenols. The free amino acid content was highest in the −20 °C treatment group (5.21 mg/g), slightly lower in the LN group (5.18 mg/g), with both being higher than the CK group (4.63 mg/g). This indicates that appropriate freezing conditions help retain the fresh and brisk substance base of the tea leaves [42]. The phenolic–amino acid ratio is an important indicator for measuring the harmony and freshness of tea soup flavor. A lower phenolic–amino acid ratio is usually associated with higher freshness and more harmonious flavor quality [43,44]. Overall, the phenol-to-amine ratio (2.111) in the −20 °C treatment group was lower than that of the control (2.147); the LN treatment group exhibited the lowest phenol-to-amine ratio (1.929), whilst the −80 °C treatment group had the highest, reaching 2.401. This is consistent with the descriptions of flavor provided in the sensory evaluation. Compared to CK, freezing treatment significantly increased the content of ECG and EGC, but the increase in EGCG was limited (highest at −20 °C), and it was not conducive to the retention of GA.

### 3.3. Effects of Different Freeze-Withering Temperature Treatments on the Aroma Components of Taiping Kuihong Black Tea

Based on the sensory evaluation results, to clarify the impact of different freezing withering temperatures on the aroma characteristics of Taiping Kuihong and to compare with CK, the HS-SPME/GC-MS method was used to identify and analyze the aroma components of the four sample groups. A total of 66 aroma compounds were detected. Among these, alcohols accounted for 54.72%, aldehydes 28.62%, esters 6.89%, terpenes 6.60%, ketones 1.13%, and other compounds 2.04%. Compared with the CK group, the frozen treatment group exhibited a significant increase in the proportions of esters and terpenes. Esters rose from 3.52% to 9.35%, while terpenes increased from 2.01% to 8.88%. Concurrently, the proportions of alcohols and aldehydes decreased. In terms of key aroma components, the frozen wilting group maintained higher levels of linalool (49.647–66.446 μg/L), methyl salicylate (40.743–91.265 μg/L), geraniol (124.430–157.686 μg/L), and benzaldehyde (43.631–64.780 μg/L). Particularly under the −20 °C treatment conditions, the average terpenoid content reached as high as 84.85 μg/L, the highest among all groups, and significantly higher than that of the LN group (28.27 μg/L) and the −80 °C group (19.13 μg/L); the average alcohol content of 281.60 μg/L was also higher than that of the LN group (200.60 μg/L) and the −80 °C group (233.94 μg/L). Meanwhile, the CK group had the highest average total aroma compound content (903.52 μg/L), while the aldehydes (310.38 μg/L) were significantly higher than in the other groups; however, the terpenes content was only 18.12 μg/L, far lower than that of the −20 °C and LN freeze-wilting groups. The results indicate that the −20 °C condition is more conducive to the conversion and accumulation of terpenoid aroma precursors; although the CK group had higher total alcohol and aldehyde content, its aroma profile was dominated by alcohols (58.63%) and aldehydes (34.35%), with both the content and proportion of terpenoids being lower than in the freeze-wilting groups.

**Figure 2:**
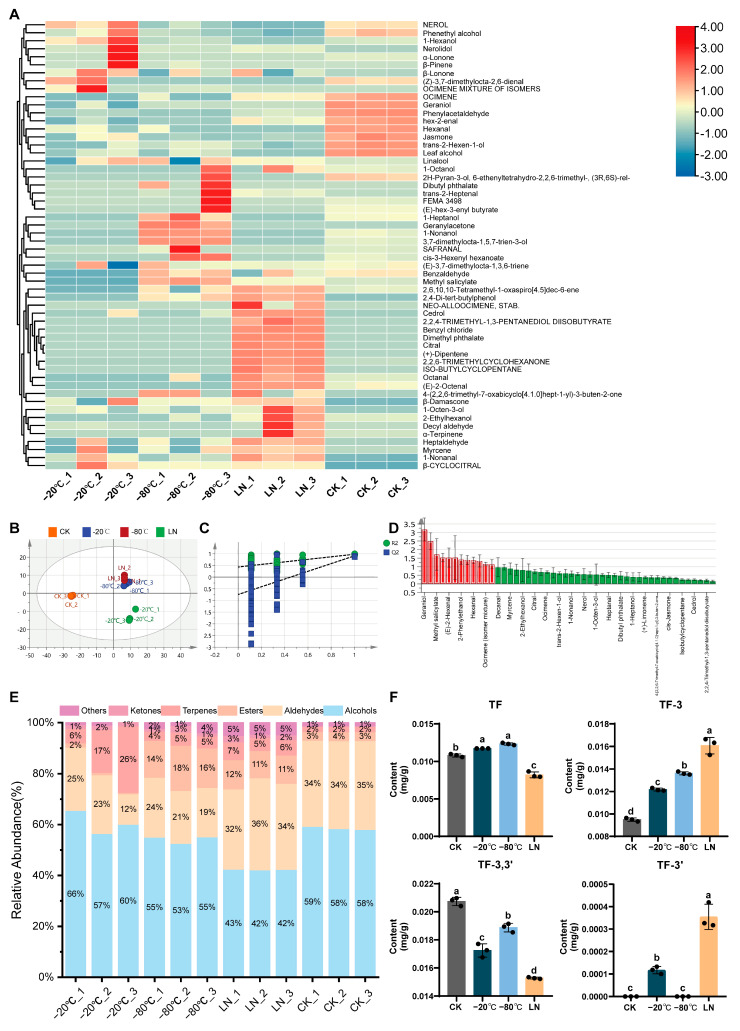
Heatmap showing the levels of volatile metabolites in different freeze-withering temperature groups and the conventional processing group (**A**); OPLS-DA analysis of different freeze-withering temperatures groups and the conventional processing group (**B**); model cross-validation results (**C**); VIP plot of differential aroma compounds in different freeze-withering temperatures groups and the conventional processing group (**D**); stacked percentage bar chart of different freeze-withering temperatures groups and traditional processing groups (**E**); bar chart of theaflavin component contents under different freeze-withering temperature groups and traditional processing groups (**F**). Note: Different lowercase letters in the same column indicate significant differences between treatments at the *p* < 0.05 level. The four treatment methods refer to −20 °C, −80 °C, liquid nitrogen treatment (−196 °C, LN), and traditional withering (CK). Abbreviations: TF, theaflavin; TF-3, theaflavin-3-gallate; TF-3′, theaflavin-3′-gallate; TF-3,3′, theaflavin-3,3′-digallate. Red-marked compounds in Figure (**D**) indicate VIP > 1.

Using 66 aroma components as the dependent variable and different freezing wilting temperatures and traditional processing methods as the independent variables, effective differentiation of the four different treatment samples was achieved through OPLS-DA (Figure 2B). In the OPLS-DA model, RX2 was 0.896, RY2 was 0.982, and Q2 was 0.921. Since both R2 and Q2 exceeded 0.5, the model fit results are acceptable [40]. The reliability of the model was verified by a 200-fold cross-validation test. The results showed that the intercept of the regression line with the vertical axis was Q2 = −0.74312 (less than zero), indicating that the OPLS-DA model possesses good predictive capability and robustness. The model validation is effective, and it is believed that this result can be used for the aroma identification analysis of four different treated samples [45].

After standardizing the relative content data of aroma compounds and creating a clustering heatmap (Figure 2A), a comparison of different freeze-withering treatment samples and CK revealed that the aroma composition of the freeze-withering treatment samples was mainly composed of alcohols, aldehydes, esters and ketones. Compared to CK, the −20 °C frozen treatment samples excelled in various key aroma components. Specifically, in the samples treated at −20 °C, the content of the alcohol compounds characteristic of floral and fruity aromas (such as nerol at 7.433 μg/L and n-hexanol at 29.169 μg/L) was significantly higher than in the LN group (2.893 μg/L, not detected) and the −80 °C group (3.951 μg/L, 5.938 μg/L), and were close to or in some cases higher than those in the CK group (6.346 μg/L, 8.763 μg/L); conversely, the content of aldehydes and alkenes exhibiting grassy or raw, green odors, such as farnesol (16.86 μg/L), was markedly lower than that in the CK group (52.24 μg/L). Furthermore, floral, fruity and woody components in the −20 °C-treated samples, such as phenethyl aldehyde (58.13 μg/L) and linalool (45.05 μg/L), also remained at relatively high levels.

From the above, it can be seen that in the cold-wilting process, treatment at −20 °C effectively preserves pleasant aromatic components such as floral, fruity and woody notes whilst significantly reducing the content of grassy compounds (*p* < 0.05). Consequently, this method yields the best aromatic quality and holds potential for further optimization and comparison with the control (CK).

### 3.4. Identification and Analysis of Aroma Component Differences in Taiping Kuihong Black Tea Under Different Freezing Wilting Temperatures

#### 3.4.1. Screening of Differential Aroma Compounds

**Figure 3:**
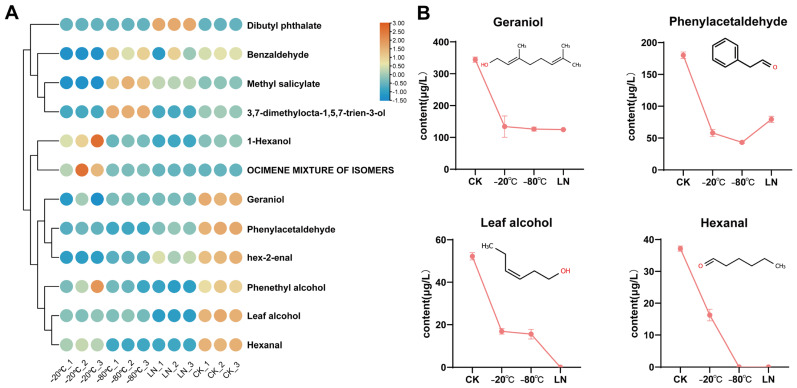
Heatmap of differential aroma compound content (**A**); bar chart of the content of four key differential aroma compounds (**B**). Note: The abbreviations shown in the figure refer to −20 °C, −80 °C, liquid nitrogen treatment (−196 °C, LN), and traditional withering (CK).

To further analyze the impact of different freezing wilting temperatures on the aroma compounds of Taiping Kuihong and the differences with CK, 12 differential aroma compounds were selected based on the criteria of *p* < 0.05 and VIP > 1 (Figure 3A), including five types of alcohols, four types of aldehydes, one type of terpene, and two types of esters. From the heatmap, in the case of substances such as a mixture of linalool isomers, methyl salicylate and 1-hexanol, the CK content was significantly lower than that observed in the freezing processes; however, the −20 °C freezing treatment group exhibited higher levels in both the mixture of linalool isomers (42.138 μg/L) and 1-hexanol (29.169 μg/L). In contrast, the content of CK in substances such as geraniol, benzaldehyde, linalool, phenylethanol, and hexanal is higher than that in the frozen group, which imparted a richer aroma to black tea. However, since linalool and hexanal typically exhibit a grassy aroma, which is not favorable for the quality of black tea aroma, their content is significantly reduced (*p* < 0.05) in the frozen treatment, resulting in a decrease in the grassy aroma.

#### 3.4.2. Analysis of the ROAV of Different Fragrances

The content of aroma components cannot be used as a basis for determining the aroma characteristics of tea; typically, it is the aroma components with high rOAV that impart the aroma characteristics to the tea [46]. Moreover, Guo et al. [47] believe that the contribution of a single aroma component to the overall aroma of tea depends on its concentration and odor threshold. Generally, the contribution of a single aroma compound to the overall aroma of tea is evaluated by calculating the rOAV. When the rOAV is greater than 1, it is considered that the aroma compound has a certain impact on the tea aroma, and when the rOAV is greater than 10, it is considered that the aroma compound makes a significant contribution to the overall aroma of the tea [48]. According to the threshold and attribute descriptions of aroma components in the literature, a total of 10 volatile compounds with rOAV > 1 were screened from the black tea samples subjected to four different withering treatments (−20 °C, −80 °C, LN, HW) (Table 5). Four compounds exhibited significant differences: phenylacetaldehyde, geraniol, leaf alcohol and n-hexanal. These volatile compounds serve as key aromatic constituents distinguishing the four withering treatments. As aroma is perceived holistically in practical sensory evaluations, it is necessary to quantify the contribution of individual volatile compounds to the overall aroma profile [49]. ACI can evaluate the contribution of specified compound to the overall aroma by calculating the ratio of the rOAV value of individual compound to the sum of rOAV [50]. Compared with CK, although the freeze-treatment group resulted in a decrease in the contribution of phenylacetaldehyde (a reduction of 66.51%) and geraniol (a reduction of 59.97%), it significantly reduced the contribution of leaf alcohols and n-hexanol—which are characteristic of the grassy scent—by as much as 84.35% and 88.75% respectively. This indicates that the freezing treatment effectively improved the aroma purity of the black tea while maximizing the preservation of its desirable flavor skeleton.

## 4. Discussion

**Figure 4:**
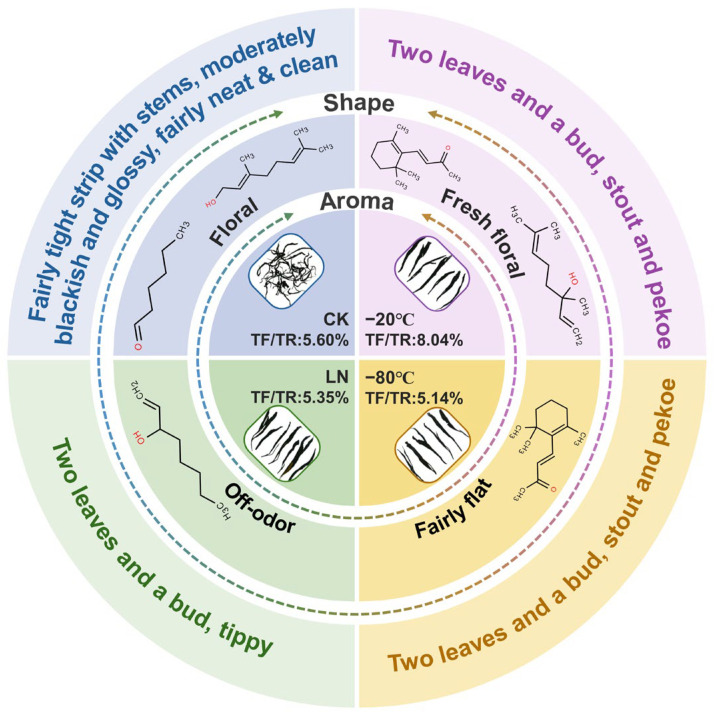
The effects of different freezing withering temperatures and traditional processing on the TF/TR ratio, volatile components, and physical appearance of Taiping Kuihong. Note: LN, liquid nitrogen treatment; CK, traditional treatment group.

This study systematically evaluated the effects of different freezing withering temperatures on the sensory quality, aroma metabolites, and main physicochemical components of Taiping Kuihong, and conducted a comprehensive comparison with CK. The results showed that the freezing withering technique demonstrated significant potential in resolving the contradiction between the shape retention and internal quality development of Taiping Kuihong black tea, but the treatment effects varied with different temper-atures (Figure 4).

### 4.1. The Freeze-Withering Treatment Promotes Cell Damage and Facilitates the Shaping of Dry Tea Leaves

In terms of maintaining appearance, all the frozen treatment groups (−20 °C, −80 °C, liquid nitrogen) of the finished tea were able to better maintain the inherent form of ‘two leaves and one bud, robust and with downy hairs,’ and their appearance sensory scores were superior to CK. Research indicates that ice crystals formed during the freezing process can cause micro-damage or even rupture to the cell walls and membrane systems, thereby increasing the permeability of the cell membranes [15,43]. This allows subsequent processes to apply gentle kneading forces that meet the requirements for mixing and contact between cell contents during the enzymatic oxidation reaction, thereby minimizing the damage to the physical structure of the tea leaves caused by traditional kneading methods. This discovery demonstrates the feasibility of the ‘frozen instead of kneading’ approach in the processing of tea products like Taiping Kuihong, which have special requirements for their shape. It effectively addressed the problem of traditional kneading easily causing shape damage and successfully filled the gap in the technical path for maintaining the shape of pointed black tea during processing.

### 4.2. The Regulation of Aroma Quality by Freeze-Withering Exhibits an Optimal Range

In shaping the aroma quality, freezing treatment played a significant role in regulation and improvement. When compared to the CK group, the freezing treatment caused significant changes in the composition of aromatic compounds: the total content of alcohols and aldehydes decreased, with a reduction in floral and fruity components such as phenethyl aldehyde and geraniol; however, the decline in grassy components such as geraniol and n-hexanal was more pronounced. At the same time, terpene concentrations increased significantly. Overall, the grassy notes were effectively reduced, resulting in a cleaner, more harmonious scent profile with a greater sensation of freshness. This is consistent with recent research indicating that freezing can enhance the scent profile of black tea [44]. In-depth analysis revealed that the −20 °C treatment performed best in terms of overall aroma quality. Under these treatment conditions, although the content of some floral and fruity aroma compounds in the tea was lower than that of CK, the content of grassy aroma compounds was effectively suppressed. This result is consistent with the conclusions of Muthumani and Senthil Kumar (2007) regarding the freeze-withering of black tea, which found that freezing treatment can influence and promote the formation or retention of certain characteristic volatile substances [51]. This study further reveals that this promoting effect exhibits significant temperature differences. In this study, treatment at −20 °C created a more suitable stress environment; whilst it effectively disrupted the cells, it may not have completely inactivated the enzyme systems involved in the conversion of aroma precursors [14], thereby facilitating the synthesis of ester compounds and the hydrolysis release of terpenoid aromas [52]. In contrast, although the quick freezing at −80 °C and with LN caused more thorough physical damage to cells, the extremely low temperatures may have led to transient inactivation of some enzyme systems or alterations in metabolic pathways, limiting the complete synthesis of specific aroma compounds. Furthermore, the rapid leakage of cell sap and subsequent non-enzymatic changes could produce unconventional off-flavors. This may be the reason why the LN sample exhibited an ‘off-flavor’ during the sensory evaluation [42].

### 4.3. Effects of Freeze-Withering Treatment on Theaflavin Content

In determining the core chemical components that affect the color and taste quality of tea, freezing treatment also shows a clear regulatory effect. The −20 °C treatment resulted in the highest content of theaflavins and the TF/TR ratio. The results of this study corroborate the previous findings [15,16,17], who all concluded that appropriate freezing wilting treatment can promote the formation of theaflavins during black tea fermentation. However, the theaflavin content in LN was the lowest among the three freezing treatments. This may be due to the need to maintain the leaves’ appearance; in this study, the tea samples subjected to freezing treatment were all lightly rolled. LN may not have fully activated key enzyme systems involved in the fermentation process, such as polyphenol oxidase, under extremely low temperatures due to insufficient rolling time and force [18,19,53]. At the same time, the tea varieties and rolling times differ from the research by Hou Binghao and others, which may be the reason for the poor performance of LN in theaflavin accumulation in this study.

### 4.4. Freeze-Withering Treatment Has a Dual Nature in Its Impact on Key Taste Components

In the freeze-withering process, not all freezing treatments are beneficial to quality. This study found that the tea polyphenol (TB) content in LN significantly increased, and excessive tea polyphenols are usually associated with a darkened liquor color and a thin taste. This may be due to the rapid freezing causing severe damage to the cell structure, combined with a short rolling time, which failed to synthesize theaflavins at a critical stage. This led to excessive and disordered oxidative polymerization of polyphenolic sub-stances during subsequent fermentation, ultimately resulting in the excessive accumulation of thearubigins. In terms of the main flavor components, the freezing treatment exhibited a common pattern: compared with CK, the phenol-to-amine ratio was generally lower in the frozen group, which may enhance freshness; however, the increase in caffeine and the decrease in soluble sugar content may result in a reduction in the sweetness and smoothness of the tea soup.

## 5. Conclusions

The results of this study indicate that freeze-withering is a new procedure that can effectively balance the preservation of Taiping Kuihong’s outward shape while improving its interior quality. Comprehensive sensory quality and non-volatile component evaluations demonstrated that −20 °C freeze-wilting resulted in the most balanced overall quality profile. This procedure not only kept the flat, straight appearance of the tea leaves, but it also greatly raised the theaflavin (TF) content and TF/TR ratio, which improved the freshness and brightness of the infusion. In terms of aroma composition, the −20 °C treatment effectively reduced the contribution of grassy notes—represented by linalool and n-hexanal—whilst maintaining a high overall aroma intensity. It also increased the relative content of compounds associated with floral, fruity and sweet aromas, such as β-ionone and linalool, resulting in a purer aroma profile. However, sensory evaluation and quantitative data also indicate that the sweetness and richness of the aroma in tea samples produced by this treatment are inferior to those of the control group processed using traditional methods. Furthermore, whilst grassy notes were reduced, the contribution of certain key aromatic compounds also decreased accordingly. Overall, freezing withering at −20 °C strikes a certain balance between aroma retention and visual quality enhancement, and may serve as a reference for subsequent research on freezing withering and the shaping of black tea. While the −80 °C treatment promoted thearubigins (TR) accumulation, it resulted in a rather monotonous scent structure and decreased theabrownin (TB) content. Conversely, liquid nitrogen treatment induced severe cellular disruption and enzyme inactivation, resulting in considerable accumulation of theabrownins (TB) and the production of off-flavors in the fragrance, lowering overall quality. This study provides preliminary evidence of the suitability of −20 °C frozen withering in the processing of Taiping Kuihong tea; however, given the limitations of a single sample batch, a single origin and specific processing details, the findings require further validation across different varieties, larger sample sizes and multiple growing regions. Future research could combine transcriptomics and metabolomic techniques to uncover important gene and enzyme activity changes in aroma and pigment manufacturing pathways under freezing stress, laying the groundwork for precise control of the freeze-withering process.

## Figures and Tables

**Table 1:** Sensory evaluation results of Taiping Kuihong tea with different freezing withering methods and traditional processing.

Tea Sample	Appearance		Liquor Color		Aroma		Taste		Spent Leaf		Total Score
	Description	Score	Description	Score	Description	Score	Description	Score	Description	Score	
−20 °C Withering (−20 °C)	Two leaves embracing a bud, sturdy and tippy	90.0 ± 0.77 a	Bright Yellow	89.0 ± 0.80 c	Fresh and pure with a sweet aroma	89.1 ± 0.83 b	Mellow and brisk	89.3 ± 0.61 a	Thick and substantial leaves, fairly even and bright with a bluish auburn color.	90.0 ± 0.92 a	89.4 ± 0.39 a
−80 °C Withering (−80 °C)	Two leaves embracing a bud, sturdy and tippy	90.1 ± 0.88 a	Bright Yellow	87.7 ± 0.70 d	Fairly pure aroma, slightly sweet	87.7 ± 0.88 c	Fairly mellow and relatively thick	87.3 ± 0.80 b	Thick and substantial leaves, fairly even and bright with a bluish auburn color.	90.1 ± 0.83 a	88.4 ± 0.43 c
Liquid Nitrogen Withering (LN)	Two leaves embracing a bud, tippy	89.1 ± 0.83 b	Orange Yellow	90.1 ± 0.70 b	With off-odor	85.7 ± 0.80 d	Relatively mellow and thick, slightly sweet	90.0 ± 0.94 a	Thick and substantial leaves, fairly even and bright with a bluish auburn color.	89.7 ± 0.88 a	88.6 ± 0.50 c
Traditional Process (CK)	Strip shape fairly tight and compact, with stems, somewhat black and glossy, fairly neat and even	89.1 ± 0.80 b	Orange Red	91.8 ± 0.56 a	Sweet aroma	90.0 ± 0.74 a	Relatively mellow and brisk	87.3 ± 0.72 b	Thick and substantial leaves, fairly even and bright with a bluish auburn color.	90.0 ± 0.74 a	89.1 ± 0.21 b

Note: Different lowercase letters in the same column indicate significant differences between treatments at the *p* < 0.05 level. The data are presented as mean ± SD (*n* = 3).

**Table 2:** Tea pigment difference table.

Method	TF (%)	TR (%)	TB (%)	TF/TR (%)
−20 °C	0.84 ± 0.01 a	10.40 ± 1.18 b	20.67 ± 0.74 b	8.04%
−80 °C	0.76 ± 0.01 b	14.70 ± 1.61 a	21.18 ± 1.04 b	5.14%
LN	0.69 ± 0.02 c	12.84 ± 2.30 ab	24.34 ± 1.43 a	5.35%
CK	0.57 ± 0.01 d	10.21 ± 0.30 b	18.06 ± 0.11 c	5.60%

Note: Different lowercase letters in the same column indicate significant differences between treatments at the *p* < 0.05 level. The data are presented as mean ± SD (*n* = 3). Abbreviations: TF, theaflavins; TR, thearubigins; TB, theabrownins.

**Table 3:** Major biochemical components of Taiping Kuihong under different freezing withering temperatures methods and traditional processing (mg/g).

Treatment	Tea Polyphenols	Soluble Sugars	Free Amino Acids	Dry Matter Content
−20 °C	11.10 ± 0.09 a	1.88 ± 0.04 b	5.21 ± 0.09 a	94.80 ± 0.08 b
−80 °C	10.75 ± 0.36 b	1.99 ± 0.01 b	4.48 ± 0.09 b	95.02 ± 0.12 b
LN	9.99 ± 0.08 c	1.85 ± 0.02 b	5.18 ± 0.17 a	93.10 ± 0.17 c
CK	9.94 ± 0.06 c	2.94 ± 0.29 a	4.63 ± 0.45 b	96.08 ± 0.13 a

Note: Different lowercase letters in the same column indicate significant differences between treatments at the *p* < 0.05 level. The data are presented as mean ± SD (*n* = 3). The different methods refer to −20 °C, −80 °C, liquid nitrogen treatment (−196 °C, LN), and traditional withering (CK).

**Table 4:** Physicochemical components of Taiping Kuihong black tea under different freezing withering temperatures and methods and traditional processing (mg/g).

Method	GA	EGC	CAF	EGCG	ECG	EC	GCG
−20 °C	0.99 ± 0.17 b	6.66 ± 0.13 b	34.82 ± 2.40 ab	8.74 ± 0.35 a	0.02 ± 0.01 a	8.18 ± 1.87 c	4.61 ± 1.45 a
−80 °C	0.76 ± 0.13 c	5.23 ± 0.14 bc	32.82 ± 1.17 b	7.26 ± 1.23 b	0.01 ± 0.001 b	18.34 ± 1.53 a	4.12 ± 0.58 ab
LN	1.11 ± 0.04 b	14.58 ± 2.42 a	36.42 ± 1.21 a	2.63 ± 0.58 c	0.02 ± 0.02 b	13.66 ± 0.35 b	2.26 ± 1.44 bc
CK	1.53 ± 0.08 a	3.44 ± 0.12 c	32.17 ± 1.43 b	2.26 ± 0.01 c	0.01 ± 0.0001 b	19.69 ± 0.12 a	1.32 ± 0.03 c

Note: Different lowercase letters in the same column indicate significant differences between treatments at the *p* < 0.05 level. The data are presented as mean ± SD (*n* = 3). The different methods refer to −20 °C, −80 °C, liquid nitrogen treatment (−196 °C, LN), and traditional withering (CK). Abbreviations: GA, gallic acid; EGC, (−)-epigallocatechin; CAF, caffeine; EGCG, (−)-epigallocatechin-3-gallate; ECG, (−)-epicatechin-3-gallate; EC, (−)-epicatechin; GCG, (−)-gallocatechin-3-gallate.

**Table 5:** Key volatile compounds and corresponding aroma descriptions of Taiping Kuihong black tea under different freezing withering treatments and traditional processing.

Compound Name	CAS Number	Threshold	Aroma Description [25]	Concentration (μg/L)				rOAV			
				−20 °C	−80 °C	LN	CK	−20 °C	−80 °C	LN	CK
Phenylacetaldehyde	122-78-1	5.2	Fruity	58.13 ± 5.64	43.28 ± 1.62	79.44 ± 4.79	180.15 ± 5.19	11.19 ± 1.08	8.32 ± 0.31	15.28 ± 1.30	34.64 ± 0.10
beta-Ionone	79-77-6	0.021	Violet	6.77 ± 2.55	3.27 ± 2.90	6.21 ± 1.21	2.54 ± 0.08	322.48 ± 121.47	293.33 ± 0.57	311.65 ± 59.50	120.95 ± 4.02
Linalool	78-70-6	0.6	Citrus, Floral	45.05 ± 27.06	63.45 ± 1.38	49.65 ± 1.74	41.32 ± 0.83	100.44 ± 10.30	106.94 ± 3.08	82.75 ± 4.09	68.87 ± 1.39
Jasmone	488-10-8	0.26	Jasmine	2.60 ± 0.94	2.96 ± 0.56	1.92 ± 0.14	4.49 ± 0.29	10.03 ± 3.62	6.88 ± 0.65	7.40 ± 0.74	17.28 ± 1.10
Nonanal	124-19-6	2.8	Citrus, Soapy	4.32 ± 1.27	1.79 ± 0.17	5.79 ± 0.52	1.70 ± 0.15	1.54 ± 0.45	1.06 ± 0.20	2.11 ± 0.20	—
Geraniol	106-24-1	3.2	Rose, Citrus	134.06 ± 33.52	126.16 ± 6.25	124.43 ± 0.13	344.20 ± 8.35	50.95 ± 1.67	39.42 ± 1.95	38.88 ± 0.06	107.56 ± 2.61
Myrcene	123-35-3	1.2	Fruity-Floral	—	11.99 ± 1.80	14.72 ± 0.76	5.71 ± 0.22	14.87 ± 2.30	10.57 ± 1.86	12.26 ± 0.89	4.76 ± 0.18
Heptanal	111-71-7	6.1	Fatty, Grassy	—	2.98 ± 0.31	5.24 ± 0.50	1.09 ± 0.07	—	—	1.12 ± 0.26	—
Leaf Alcohol	928-96-1	3.9	Grassy	16.86 ± 1.34	15.56 ± 2.26	—	52.24 ± 1.70	4.32 ± 0.49	3.57 ± 0.16	—	13.39 ± 0.44
n-Hexanal	66-25-1	2.4	Grassy, Apple	16.29 ± 1.83	—	—	37.16 ± 0.82	6.79 ± 0.76	7.84 ± 0.33	—	15.48 ± 0.34

Note: Data are presented as mean ± SD (*n* = 3). Abbreviations: LN, liquid nitrogen treatment; CK, traditional treatment group. Odor thresholds (OTs) in water from: Leibniz-LSB@TUM Odorant Database (https://www.leibniz-lsb.de/en/, accessed on 16 October 2025) and (Zhai et al., 2022 [48]).

## Data Availability

The raw data supporting the conclusions of this article will be made available by the authors on request.
